# Modified FOLFOX-6 chemotherapy in advanced gastric cancer: Results of phase II study and comprehensive analysis of polymorphisms as a predictive and prognostic marker

**DOI:** 10.1186/1471-2407-8-148

**Published:** 2008-05-27

**Authors:** Bhumsuk Keam, Seock-Ah Im, Sae-Won Han, Hye Seon Ham, Min A Kim, Do-Youn Oh, Se-Hoon Lee, Jee Hyun Kim, Dong-Wan Kim, Tae-You Kim, Dae Seog Heo, Woo Ho Kim, Yung-Jue Bang

**Affiliations:** 1 Department of Internal Medicine, Seoul National University Hospital, Seoul, South Korea; 2 Cancer Research Institute, Seoul National University College of Medicine, Seoul, South Korea; 3 Department of pathology, Seoul National University Hospital, Seoul, South Korea

## Abstract

**Background:**

The objective of this study was to evaluate the efficacy and toxicity of infusional 5-fluorouracil (5-FU), folinic acid and oxaliplatin (modified FOLFOX-6) in patients with advanced gastric cancer (AGC), as first-line palliative combination chemotherapy. We also analyzed the predictive or prognostic value of germline polymorphisms of candidate genes associated with 5-FU and oxaliplatin.

**Methods:**

Seventy-three patients were administered a 2 hour infusion of oxaliplatin (100 mg/m^2^) and folinic acid (100 mg/m^2^) followed by a 46 hour continuous infusion of 5-FU (2,400 mg/m^2^). Genomic DNA from the patients' peripheral blood mononuclear cells was extracted. Ten polymorphisms within five genes were investigated including TS, GSTP, ERCC, XPD and XRCC.

**Results:**

The overall response rate (RR) was 43.8%. Median time to progression (TTP) and overall survival (OS) were 6.0 months and 12.6 months, respectively. Toxicities were generally tolerable and manageable. The RR was significantly higher in patients with a 6-bp deletion homozygote (-6 bp/-6 bp) in TS-3'UTR (55.0% *vs*. 30.3% in +6 bp/+6 bp or +6 bp/-6 bp, *p *= 0.034), and C/A or A/A in XPD156 (52.0% *vs*. 26.1% in C/C, *p *= 0.038). The -6 bp/-6 bp in TS-3'UTR was significantly associated with a prolonged TTP and OS. In a multivariate analysis, the 6-bp deletion in TS-3'UTR was identified as an independent prognostic marker of TTP (hazard ratio = 0.561, *p *= 0.032).

**Conclusion:**

Modified FOLFOX-6 chemotherapy appears to be active and well tolerated as first line chemotherapy in AGC patients. The 6-bp deletion in TS-3'UTR might be a candidate to select patients who are likely to benefit from 5-FU based modified FOLFOX-6 in future large scale trial.

## Background

Despite improvements in the early detection of gastric cancer, a significant proportion of patients present with inoperable stages where chemotherapy is required. 5-fluorouracil (5-FU) remains the main chemotherapeutic agent for the treatment of gastric cancer, and combination chemotherapy with 5-FU has shown an improved clinical outcomes [1]. 5-FU with cisplatin showed an effective clinical outcome [2], however, toxicities were considerable [1]. Oxaliplatin, another platinum based agent, has a more favorable tolerability profile than cisplatin. Hence, a combination chemotherapy of 5-FU with oxaliplatin has been investigated in numerous phase II studies, using different doses and schedules [3-7]. However it remains to be clarified which is the best combination, with the highest efficacy and lowest toxicity. Thus, we conducted a phase II trial of 5-FU, folinic acid and oxaliplatin (a modified FOLFOX-6 regimen) in advanced gastric cancer (AGC) patients as a first line palliative chemotherapy.

Another problem in chemotherapy of AGC is the selection of patients who might benefit from specific chemotherapy. One promising therapeutic challenge is to identify genetic markers based on pharmacogenomics. Genomic polymorphism can influence drug transport, metabolism and cellular response, and lead to individual variations in terms of the response and toxicity and even to overall survival [8,9]. A number of studies have investigated the relationships between treatment outcomes and individual genetic polymorphisms which will determine the efficacies and toxicities of chemotherapeutic agents, especially of 5-FU and platinum agents.

The antitumor effect of 5-FU has ascribed to the competitive inhibition of thymidylate synthase (TS) [10]. A high intratumoral TS expression has been correlated with resistance to 5-FU and a poor clinical outcome in colorectal cancer [11-14]. Several polymorphisms in TS may influence TS mRNA transcription, stability, or protein expression. Polymorphisms with double or triple repeats of a 28-base pair (bp) sequence in the enhancer region (ER) are known to be associated with the efficacy and toxicity of 5-FU [15-17]. The -6 bp/-6 bp deletion polymorphism in the 3'UTR of TS is associated with decreased mRNA stability in vitro and lower intratumoral TS expression *in vivo*. Further, the 6 bp polymorphism varies greatly within different ethnic populations and is in linkage disequilibrium with the TS 5' tandem repeat enhancer polymorphism [18]. A functional G/C single nucleotide polymorphism (SNP) within a second repeat of triple repeat (3R) allele was found to determine two additional alleles (3G or 3C) at this locus [19]. *In vitro*, the 3G containing genotype showed a higher TS mRNA expression [19,20].

Oxaliplatin has antitumor activity by virtue of its ability to form platinum-DNA adducts. Bulky platinum-DNA adducts are mainly repaired by the nucleotide excision repair pathway, in which proteins of the excision repair cross-complementation 1 (ERCC1), xeroderma pigmentosum group D (XPD, also known as ERCC2) and X-ray repair cross-complementing group (XRCC), have important roles [13,21]. ERCC, XPD and XRCC contain SNPs that may confer different activities to platinum agents, thus modifying the clinical outcome [22-24]. Glutathione S- transferase π 1 (GSTP1), which is involved in platinum detoxification, also has a polymorphism that is associated with prolonged survival in cisplatin-treated gastric cancer [25,26].

The primary endpoint of this study was to evaluate the efficacy in terms of response rate and the secondary endpoints of this study were to evaluate the efficacy in terms of time to progression, overall survival and toxicity of modified FOLFOX-6 chemotherapy in AGC patients. Exploratory pharmacogenomic collateral study was performed to identify the predictive or prognostic value of germline polymorphisms of candidate genes associated with 5-FU and oxaliplatin.

## Methods

### Patients

Patients with metastatic or relapsed AGC were enrolled in this prospective phase II clinical trial. Patients were administered a modified FOLFOX-6 regimen composed of a 2 hours infusion of oxaliplatin (100 mg/m^2^) and folinic acid (100 mg/m^2^), followed by a 46 hour continuous infusion of 5-FU (2,400 mg/m^2^), as a first-line palliative chemotherapy. Treatment was repeated every 2 weeks until disease progression, patient refusal or unacceptable adverse reactions.

Eligibility criteria included: 1) pathologically confirmed gastric adenocarcinoma with a bi-dimensionally measurable lesion; 2) no prior chemotherapy except adjuvant chemotherapy administered more than 6 months previously; 3) ECOG performance 0–2; 4) adequate bone marrow, hepatic, and renal functions. ECOG performance status [27] defined as follows: ECOG 0-fully active, able to carry on all pre-disease performance without restriction; ECOG 1-restricted in physically strenuous activity but ambulatory and able to carry out work of a light or sedentary nature; ECOG 2 – ambulatory and capable of all self care but unable to carry out any work activities; ECOG3 – capable of only limited self care, confined to bed or chair more than 50% of waking hours; ECOG 4 – completely disabled.

Patients received a blood test for toxicity every cycle and were re-evaluated every 3 cycles by abdominal computed tomography. The tumor responses were evaluated using WHO criteria [28] and all responses were confirmed at least 4 weeks after initial assessment. Toxicity was graded according to National Cancer Institute common terminology criteria for adverse events (CTCAE version 3.0). Peripheral sensory neuropathy was also graded according to same toxicity criteria. In the event of toxicity, dose modification and treatment delays were performed according to the protocol. Dose modification of oxaliplatin to 85 mg/m^2 ^was planned if the patient experienced grade 2 or 3 sensory neuropathy and we strictly followed the protocol which permitted the initiation of chemotherapy after recovery from all toxicities less than grade 2.

### Genotyping

To evaluate the clinical usefulness of germline genotyping, we analyzed peripheral blood samples that were obtained from patients with informed consent. Genomic DNA was extracted from peripheral blood samples using QIAmp DNA blood kits (Qiagen Inc, Valencia, CA, USA), and each polymorphism was analyzed using polymerase chain reaction-restriction fragment length polymorphism methods. Table 1 summarizes the primer sequences, the restriction enzymes used [15,16,19,29-34]. The polymorphisms investigated included TS (28-bp repeat in enhancer region [16], G/C SNP in the 3R allele [19], a 6-bp deletion in 3'UTR [15]), GSTP1 (Ile105Val [29]), ERCC1 (Asn118Asn [30], C8092A [31]), XPD (Arg156Arg [32], Asp312Asn [33], Lys751Gln [33]), and XRCC (Arg399Gln [34]).

**Table 1:** Primer sequences and restriction enzymes

Gene	Polymorphisms	Location	Primer	Restriction enzyme	References
TS (Ch 18p11.32)	2R or 3R VNTR in ER*	ER (5'UTR)	Forward: GTGGCTCCTGCGTTTCCCCC		16
			Backward: GCTCCGAGCCGGCCACAGGCATGGCGCGG		
	G/C SNP in 3R	5'UTR	Forward:: GTGGCTCCTGCGTTTCCCCC	Hae III	19
			Backward: GCTCCGAGCCGGCCACAGGCATGGCGCGG		
	6 bp insertion(+)/deletion(-)	3'UTR	Forward: CAAATCTGAGGGAGCTGAGT	Dra I	15
			Backward: CAGATAAGTGGCAGTACAGA		
GSTP1 (Ch.11q13)	A/G, Ile105Val	Exon5	Forward: CTCTATGGGAAGGACCAGCA	BsmA I	29
			Backward: TGAGGGCACAAGAAGCCCCT		
ERCC1 (Ch.19q13.2)	C/T, Asn118Asn	Exon4	Forward: TCATCCCTATTGATGGCTTCTGCCC	BsrD I	30
			Backward: GACCATGCCCAGAGGCTTCTCATAG		
	C8092A	3'UTR	Forward: CAGAGACAGTGCCCCAAGAG	Mbo II	31
			Backward: GGGCACCTTCAGCTTTCTTT		
XPD (Ch.19q13.3)	C/A, Arg156Arg	Exon6	Forward: CACACCTGGCTCATTTTTGTAT	Tfi I	32
			Backward: TCATCCAGGTTGTAGATGCCA		
	G/A, Asp312Asn	Exon10	Forward: CTGTTGGTGGGTGCCCGTATCTGTTGGTCT	Sty I	33
			Backward: (TAATA)TCGGGGCTCACCCTGCAGCACTTCCT		
	A/C, Lys751Gln	Exon23	Forward: GCCCGCTCTGGATTATACG	Pst I	33
			Backward: CTATCATCTCCTGGCCCCC		
XRCC (Ch.19q13.2)	G/A, Arg399Gln	Exon10	Forward: TTGTGCTTTCTCTGTGTCCA	Msp I	34
			Backward: TCCTCCAGCCTTTTCTGATA		

Ch, chromosome; VNTR, variable number tandem repeats; UTR, untranslated region; ER, enhancer region; TS, thymidylate synthase; GSTP, glutathione S- transferase π; ERCC1, excision repair cross-complementation 1; XPD, xeroderma pigmentosum group D; XRCC, X-ray repair cross-complementing group.

*VNTR polymorphism is a double repeat (2R) or triple repeat (3R) of 28-bp sequence in TS 5'UTR.

The study protocol was reviewed and approved by the institutional review board of Seoul National University Hospital.

### Statistical analyses

A single-stage design by A'Hern is chosen for definition of the total number of patients required for the phase II study [35]. We set response rate of 35% as the target activity level [6] and chose 20% as the lowest response rate. Our study is designed to have 90% power to accept the hypothesis and 5% significance to reject the hypothesis. In this phase II study, at least 73 evaluable patients will be required to be enrolled. Associations between response rate and polymorphism were assessed by a Chi-square test and Fisher's exact test, where appropriate. To verify allelic frequencies, a Chi-square test was used to confirm agreement with the Hardy-Weinberg equilibrium. Time to progression (TTP) was defined as the interval between the initiation of treatment and the date when disease progression was first documented, or the date of death from any cause. Overall survival (OS) was measured from the date of treatment initiation to the date of death. Median TTP and OS according to prognostic factors were calculated using the Kaplan-Meier method. Multivariate analyses were carried out using Cox proportional hazard regression models for TTP and OS. All values were two sided and statistical significance was accepted at the *p *< 0.05 level. SPSS version 12.0 software (SPSS, Inc., Chicago, IL, USA) was used for all statistical analyses.

### Cell culture and 5-FU sensitivity

Seven human gastric cancer cell lines (SNU 1, 5, 16, 484, 601, 620 and 638, obtained from the Korean Cell Line Bank, Seoul, Korea) were examined. Genotypes of each cell line were determined by same methods as described above. Equal numbers of cells harvested during the exponential growth phase were plated in 100 μl per well with various concentrations (0.1 nM to 50 μM, diluted in semilog fashion) for 72 hours. The growth inhibition was assessed using the 3-(4,5-dimethylthiazol-2-yl)-2,5-diphenyltetrazolium bromide test (MTT test; Sigma, St Louis, MO, USA) and the concentrations required to inhibit cell growth by 50% (IC_50_) were calculated.

### Western blot

TS protein expressions were determined by Western immunoblotting, as previously described [36]. Equivalent amounts of protein were processed by 12% SDS-PAGE, and electroblotted on to Hybond ECL nitrocellulose membranes, which were blocked overnight using blocking buffer, and then incubated with primary antibody against human TS (monoclonal mouse anti-TS antibody 1:1000 dilution in blocking buffer; TS Ab-1, clone TS106, Neomarkers). Proteins were detected by enhanced chemiluminescence. For the semiquantitative analysis of Western blots, subsaturated autoradiograms were scanned and signals were analyzed by densitometry (TINA 2.09 software; Raytest, Straubenhardt, Germany).

## Results

### Patient characteristics and clinical outcomes

Eighty patients were included in this trial and 7 patients were lost to follow up. A total of 73 patients, composed of 48 men and 25 women, with a median age of 59 years (range: 24–77) were finally evaluated in the study which ran from March 2003 to March 2005. The clinical characteristics of patients are summarized in Table 2. During a median follow-up of 21.2 months, 42 death events and 68 progression events occurred. A total of 470 cycles of modified FOLFOX-6 were delivered with a median number of 6 cycles per patient (range, one to 12). Dose intensity level was 83.2%. The median cumulative dose for oxaliplatin was 570 mg/m^2^. The overall response rate (RR) was 43.8% (Table 3). The median TTP and OS were 6.0 months (95% CI, 4.8–7.2 months) and 12.6 months (95% CI, 8.7–16.5 months), respectively. Only performance status was found to be a statistically significant prognostic factor for TTP (6.3 months in ECOG 0–1 *vs*. 3.7 months in ECOG 2, *p *= 0.037). RR, TTP and OS were not different according to histotype (diffuse *vs*. intestinal type). Otherwise no significant association was observed between patient characteristics and clinical outcomes.

**Table 2:** Patient characteristics

Characteristics	No. of Pts (N = 73)	%
Sex		
Male	48	65.8
Female	25	34.2
Age, years		
median	59	
range	24–77	
Performance status		
ECOG 0–1	60	82.2
ECOG 2	13	17.8
Histopathologic type		
Intestinal type	47	64.4
Diffuse type	23	31.5
Unknown	3	4.1
Disease status		
Relapsed	19	26.0
Initial stage IV	54	74.0
Metastatic site		
Liver	30	41.1
Peritoneum	29	39.7
LN (distant M1 node)	23	31.5
Others	18	24.7

ECOG, eastern cooperative oncology group; LN, lymph node.

**Table 3:** Results of modified FOLFOX-6 chemotherapy

Response	No. of Pts (%)
Complete response	0 (0.0)
Partial response	32 (43.8)
Stable disease	21 (28.8)
Progressive disease	20 (27.4)

### Toxicities

Severe hematologic toxicities were uncommon (Table 4). The main grade 3 and 4 toxicity (per patients) was neutropenia (11.0%). Non-hematologic toxicities were mild and 1.4% experienced grade 3 peripheral neuropathy. No febrile neutropenia was observed. Toxicities were generally brief, reversible and manageable.

**Table 4:** Toxicities according to National Cancer Institute CTCAE (per patient)

Toxicities	Grade 1–2		Grade 3–4	
		
	No. of Pts	%	No. of Pts	%
Hematologic				
Leucopenia	13	17.8	0	0.0
Neutropenia	10	13.7	8	11.0
Anemia	12	16.4	0	0.0
Thrombocytopenia	5	6.8	1	1.4
Non-hematologic				
Neuropathy	12	16.4	1	1.4
AST/ALT abnormality	3	4.1	0	0.0
Nausea	19	26.0	0	0.0
Vomiting	9	12.4	0	0.0
Mucositis	1	1.4	1	1.4

AST, Aspartate aminotransferase; ALT, Alanine aminotransferase.

### Correlation between genotypes and response rates

We analyzed 10 germline polymorphisms within 5 genes. All observed genotype frequencies were in agreement with the Hardy-Weinberg equilibrium. The genotype distributions of -6 bp/-6 bp, +6 bp/-6 bp and +6 bp/+6 bp were observed in 40 (54.8%), 30 (41.1%) and 3 (4.1%), respectively. The frequencies of 3G containing genotype and non-3G containing genotype were 69.9% and 28.8%, respectively. Table 5 compares RR and polymorphisms. RR was significantly higher in patients with a -6 bp/-6 bp homozygote in TS-3'UTR (55.0% *vs*. 30.3% in +6 bp/+6 bp or +6 bp/-6 bp, *p *= 0.034). There was no difference in RR according to TS enhancer region polymorphism (47.1% in 3G containing genotype *vs*. 33.3% in non-3G containing genotype, *p *= 0.285). In XPD Arg156Arg, a higher RR was observed in C/A or A/A genotypes compared with the C/C genotype (52.0% *vs*. 26.1%, *p *= 0.038). The other polymorphisms were not found to be significantly associated with RR. No association was observed between polymorphisms and toxicities (data not shown).

**Table 5:** Response rate according to the clinical factors and the genotypes

		Overall frequency		Responder		
				
		No. of Pts	%	No. of Pts	RR (%)	*p*-value*
Age	≤ 55	31	42.5	15	48.4	0.501
	> 55	42	57.5	17	40.5	
Performance	ECOG 0–1	60	82.2	28	46.7	0.295
	ECOG 2	13	17.8	4	30.8	
Histotype	Intestinal	47	67.1	19	40.4	0.557
	Diffuse	23	32.9	11	47.8	
	unknown	3				
Disease status	Initial stage IV	54	74.0	27	50.0	0.107
	Relapsed	19	26.0	5	26.3	
TS in 5'UTR^*#*^	2R/2R, 2R/3C, 3C/3C	21	28.8	7	33.3	0.285
	2R/3G, 3C/3G, 3G/3G	51	69.9	24	47.1	
	unknown	1	1.4			
TS 6-bp deletion in 3'UTR	-6/-6	40	54.8	22	55.0	0.034
	+6/+6 or +6/-6	33	45.2	10	30.3	
GSTP1-Ile105Val (A105G)	A/A	44	60.3	22	50.0	0.191
	A/G or G/G	29	39.7	10	34.5	
ERCC-Asn118Asn	C/C	40	54.8	17	42.5	0.800
	C/T or T/T	33	45.2	15	57.5	
ERCC-C8092A	C/C	44	60.3	18	40.9	0.535
	C/A or A/A	29	39.7	14	48.3	
XPD- Arg156Arg	C/C	23	31.5	6	26.1	0.038
	C/A or A/A	50	54.0	26	52.0	
XPD-Asp312Asn	G/G	8	11.0	1	12.5	0.072
	G/A	65	89.0	31	47.7	
XPD-Lys751Gln	A/A	62	84.9	28	45.2	0.746
	A/C or C/C	11	15.1	4	36.4	
XRCC1-Arg399Gln	G/G	48	65.8	21	43.8	0.984
	G/A or A/A	25	34.2	11	44.0	

RR, response rate.

*p-value for the comparison between response rate and genotypes, based on Pearson's χ 2 test (using Fisher's exact test, if N ≤ 5).

^#^Analysis of the TS-5'UTR VNTR polymorphism with G/C SNP change in 3R allele carriers.

### Time to progression, overall survival, and the correlation with genotype

**Figure 1:**
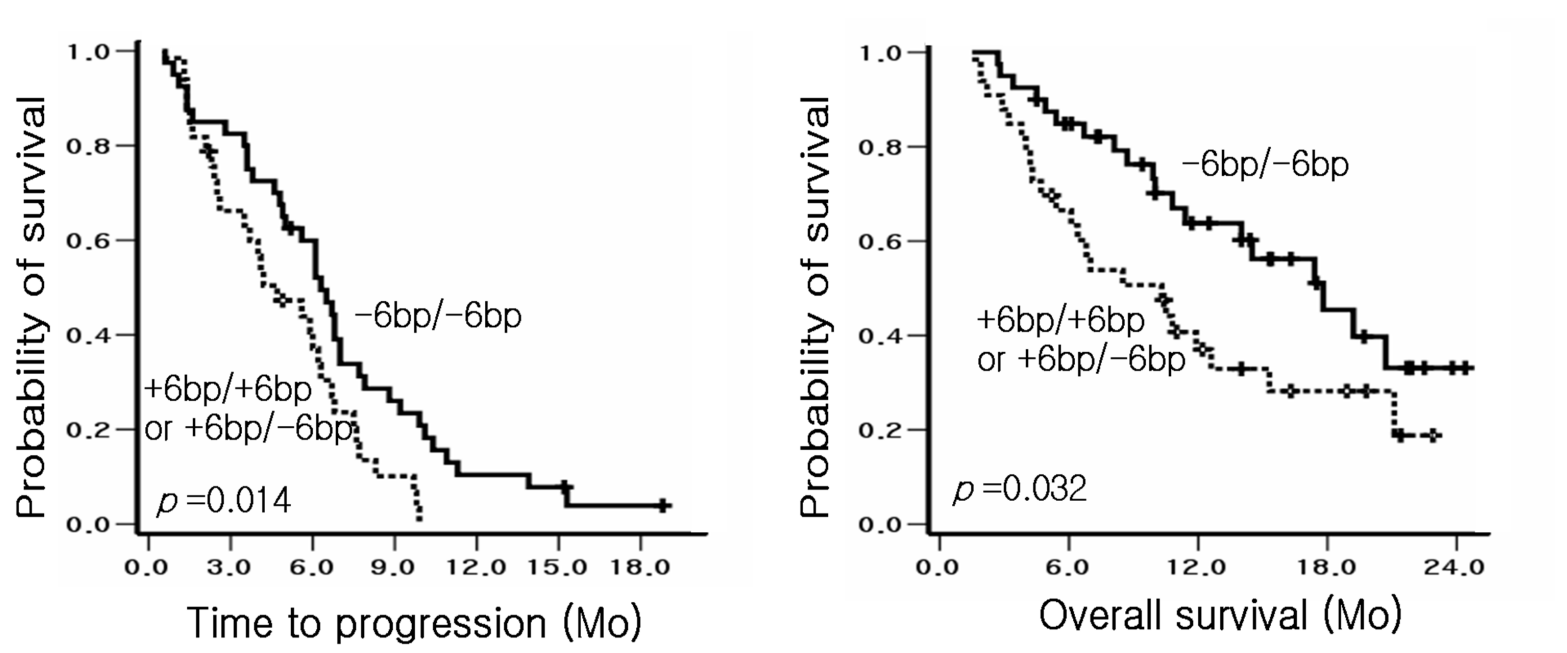
**Kaplan-Meier plots of TTP and OS, according to 6-bp deletion polymorphism in 3'UTR of TS.** The 6-bp deletion homozygote (-6 bp/-6 bp) was significantly associated with prolonged TTP (6.3 months *vs*. 4.7 months in +6 bp/+6 bp or +6 bp/-6 bp, *p *= 0.014) and OS (17.8 months vs. 10.3 months in +6 bp/+6 bp or +6 bp/-6 bp, *p *= 0.032).

Among the polymorphisms investigated, only the 6-bp deletion homozygote (-6 bp/-6 bp) in TS-3'UTR was found to be significantly associated with a favorable TTP and OS. The TTP of patients with -6 bp/-6 bp in TS-3'UTR was 6.3 months, whereas that of patients with +6 bp/+6 bp or +6 bp/-6 bp was 4.7 months (*p *= 0.014, *p*-value based on log rank test), and a significant difference was also observed between these genotypes in terms of OS (17.8 months in -6 bp/-6 bp *vs*. 10.3 months in +6 bp/+6 bp or +6 bp/-6 bp, *p *= 0.032, *p*-value based on log rank test). In Figure 1 Kaplan-Meier plots are shown and reveal the relationship between TTP and OS according to the TS-3'UTR polymorphism. In addition, a prolonged TTP was observed in the C/A or A/A genotype of XPD Arg156Arg (6.2 months *vs*. 4.1 months in C/C, *p *= 0.022). However, no association was observed for the other polymorphisms and TTP or OS (Table 6). Variables showing association with TTP in univariate analysis with *p *< 0.10 (6-bp deletion in TS-3'UTR, XPD- Arg156Arg, XPD-Asp312Asn, performance status) were included for multivariate analysis using Cox proportional hazard regression models. In multivariate analysis, TS-3'UTR was identified as an independent prognostic marker of survival (Table 7). The 6-bp deletion in TS-3'UTR was independently associated with a prolonged TTP. (Hazard Ratio [HR] = 0.561).

**Table 6:** Comparison of TTP and OS according to the clinical factors and genotypes

		Time to progression				Overall survival			
			
		Median TTP (Mo)*	HR^#^	*95% *CI	*p*-value^#^	Median OS (Mo)*	HR^#^	*95% *CI	*p*-value^#^
Age	≤ 55	5.6	1.000		0.485	11.9	1.000		0.146
	> 55	5.0	0.840	0.514–1.371		10.8	1.610	0.847–3.060	
Performance	ECOG 0–1	6.0	1.000		0.042	12.6	1.000		0.124
	ECOG 2	3.7	1.892	1.022–3.501		10.0	1.722	0.862–3.441	
Histotype	Intestinal	6.1	1.000		0.495	14.5	1.000		0.392
	Diffuse	5.6	1.197	0.714–2.009		10.8	1.337	0.687–2.602	
Disease status	Initial stage IV	5.6	1.000		0.362	12.6	1.000		0.903
	Relapsed	6.0	1.288	0.748–2.219		10.8	0.959	0.489–1.881	
TS 5'UTR	2R/2R, 2R/3C, 3C/3C	5.6	1.000		0.781	10.8	1.000		0.244
	2R/3G, 3C/3G, 3G/3G	6.1	0.781	0.415–1.470		17.4	0.669	0.340–1.316	
TS 6-bp deletion in 3'UTR	+6/+6 or +6/-6	4.7	1.000		0.033	10.3	1.000		0.046
	-6/-6	6.3	0.572	0.342–0.956		17.8	0.536	0.291–0.988	
GSTP1-Ile105Val	A/A	6.0	1.000		0.398	11.9	1.000		0.621
	A/G or G/G	6.3	1.243	0.750–2.061		14.5	0.621	0.452–1.606	
ERCC-Asn118Asn	C/C	6.2	1.000		0.433	14.5	1.000		0.472
	C/T or T/T	5.0	0.822	0.503–1.342		11.9	1.251	0.680–2.302	
ERCC-C8092A	C/C	6.1	1.000		0.826	17.8	1.000		0.326
	C/A or A/A	5.6	0.945	0.569–1.568		11.4	1.356	0.739–2.490	
XPD- Arg156Arg	C/C	4.1	1.000		0.022	10.5	1.000		0.355
	C/A or A/A	6.2	0.533	0.311–0.913		14.5	0.738	0.367–1.405	
XPD-Asp312Asn	G/G	6.1	1.000		0.084	14.0	1.000		0.673
	G/A	3.5	1.942	0.914–4.129		10.8	0.800	0.283–2.256	
XPD-Lys751Gln	A/A	6.1	1.000		0.693	12.6	1.000		0.173
	A/C or C/C	4.6	0.872	0.440–1.725		Not reached	0.488	0.174–1.369	
XRCC1-Arg399Gln	G/G	6.1	1.000		0.744	15.3	1.000		0.160
	G/A or A/A	5.9	1.090	0.649–1.832		10.3	1.575	0.835–2.971	

Mo, months; HR, hazard ratio; CI, confidence interval.

* By Kaplan-Meier analysis

^#^By Cox proportional hazard regression model, adjusted for performance

Note: If the hazard ratio is greater than 1, the hazard ratio can be thought of as the average increased risk of progression, or death at any point in time, compared with the reference group (described above line).

**Table 7:** Clinical factors and genotypes influencing survival in multivariate analyses

	TTP		OS	
		
	HR (95%CI)	*p*-value	HR (95%CI)	*p*-value
TS 6-bp deletion in 3'UTR	0.561 (0.331–0.950)	0.032	0.553 (0.285–1.072)	0.079
XPD-Arg156Arg	0.705 (0.407–1.220)	0.210	0.923 (0.456–1.867)	0.823
XPD-Asp312Asn	1.956 (0.902–4.240)	0.089	0.802 (0.281–2.286)	0.679
Performance (ECOG 0–1 *vs*. 2)	1.973 (1.034–3.762)	0.073	1.638 (0.816–3.288)	0.165

### Correlations between in vitro data and the TS polymorphism

The above clinical result led to further investigation for theoretical confirmation in *in vitro *condition. Using seven human gastric cancer cell lines and Western blotting, we investigate for correlations between genotypes, protein expressions, and 5-FU sensitivities. Two out of seven cell lines (SNU 601, 620) had -6 bp/-6 bp homozygotes polymorphism in TS 3'UTR. Six bp deletion homozygotes tended to show lower TS protein expression (TS/tubulin expression ratio 0.88 in -6 bp/-6 bp *vs*. 1.15 in +6 bp/+6 bp or +6 bp/-6 bp, *p *= 0.095 by Mann-Whitney U test). Subsequent Western blot analysis showed lower TS expression was associated with higher sensitivity to 5-FU in our *in vitro *data [36]. Linear regression analysis revealed a statistically significant correlation between TS protein expression and the IC_50 _of 5-FU (*p *= 0.002, r^2 ^= 0.887), and the 6-bp deletion polymorphism in TS-3'UTR was associated with lower TS expression and more sensitivity to 5-FU in gastric cancer cell lines *in vitro*.

## Discussion

In this work we evaluated the efficacy of modified FOLFOX-6 chemotherapy in AGC patients, and examined the relevance of the relationship between germline genetic polymorphisms and the clinical outcome. The results indicate that a modified FOLFOX-6 chemotherapy appears to be active and well tolerated.

With an overall RR of 43.8%, our results compare favorably with other phase II studies of FOLFOX chemotherapy, which range from 38% to 56% [3-7]. By contrast to the FOLFOX-6 regimen for AGC [3], the regimen here omitted the 5-FU bolus injection in order to reduce myelosuppression. In terms of toxicities, the modified FOLFOX-6 regimen showed an 11.0% occurrence of grade 3 or 4 neutropenia, which is lower than the 38% level shown with the FOLFOX-6 regimen [3]. Grade 3 or 4 peripheral sensory neuropathy occurred in only 1.4% of the patients. This was lower than original FOLFOX-6 using oxaliplatin of 100 mg/m^2 ^with a median cumulative dose of 901 mg/m^2 ^for oxaliplatin. In our study the median cumulative dose of 570 mg/m^2 ^for oxaliplatin which is lower than original FOLFOX-6. With considering median cumulative dose of oxaliplatin, this was comparable to other lower dose oxaliplatin-based regimen or omitting the 5-FU bolus [4,7]. In our study, dose modification of oxaliplatin to 85 mg/m^2 ^was performed if the patient experienced grade 2 peripheral neuropathy and we strictly followed the protocol which permitted the initiation of chemotherapy after recovery from all toxicities less than grade 2.

In the present study, TS and XPD polymorphisms were found to be associated with RR, and these polymorphisms could be used as predictive markers. TS, the target of 5-FU, has been investigated repeatedly in various aspects to predict the response to 5-FU, in terms of polymorphism, mRNA and protein expressions [10,16]. In our study, the 6-bp deletion polymorphism in TS-3'UTR was found to be correlated with favorable clinical outcome. That means that the patients with -6 bp/-6 bp were found to have a higher RR and prolonged time to progression. TS-3'UTR plays a role as a post transcriptional regulator, mainly by controlling mRNA stability and/or translational efficiency. The 6-bp deletion in TS-3'UTR reduces mRNA stability and lowers intratumoral TS mRNA levels [18]. A reduced level of TS mRNA might also lead to a lower TS protein expression, and make tumors more sensitive to 5-FU based chemotherapy [13,36,37]. Previous studies in colorectal cancer provide strong evidence that a lower TS expression appears to be associated with a higher RR and prolonged survival with 5-FU based chemotherapy [11,13,14]. These findings are supported by *in vitro *data that -6 bp/-6 bp in TS-3'UTR affect to lower TS expression and higher sensitivity to 5-FU in gastric cancer cell lines. In gastric cancer, we confirmed that a 6-bp deletion is associated with better clinical outcome in homogenous patients treated with a first line modified FOLFOX-6 regimen.

However, some studies conducted in Western countries [25,26] failed to find a correlation between the 6-bp deletion polymorphism and 5-FU sensitivity in gastric cancer, while our results are concordant with those of another study conducted in an Asian [38]. One possible explanation for this contradictory result is ethnic difference.

Caucasian patients had higher proportion of favorable 2R/2R genotype (16.0% to 26.4%) [26,39,40], lower proportion of unfavorable 3R/3R or 3G containing genotypes in 5'UTR (28.8% to 39.2%) [26,34,40] and lower proportion of favorable -6 bp/-6 bp in 3'UTR (7.8% to 22.0%) [18,26,34,40]. In contrast, Asian patients showed lower proportion of favorable 2R/2R (0.0% to 6.0%) [34,41] higher proportion of unfavorable 3R/3R (66.4% to 72.3%) [34,42] and higher proportion of favorable -6 bp/-6 bp (36.4% to 56.0%) [18,34,38]. These differences between favorable and unfavorable genotype frequencies might make different results according to ethnic diversity.

The clinical influence of TS polymorphism in 5-FU based chemotherapy are often controversial because of different clinical settings (adjuvant *vs*. palliative), tumor type (colon *vs*. gastric cancer), and different 5-FU infusion times [16,25,26,39,42-45]. These experimental variables should be considered when interpreting predictive values of the TS polymorphism.

Another factor to be taken into consideration is the possible impact on outcome of other drugs combined with 5-FU. Even antitumor activity of 5-FU may be decreased by TS polymorphism, another combined drug might compensate the decreased antitumor activity and make sufficient tumor response. This would make the meaningful polymorphism in single agent protocol less significant in combination regimens [46]. Thus this could account for the differing results with prior reports. A meta-analysis could perhaps provide some clarity in this area.

XPD (also known as ERCC2) encodes DNA helicase, which is a member of the nuclear excision repair pathway and plays a role in repairing platinum-DNA adducts. The C/C genotype of XPD-Arg156Arg SNP showed lower RR than C/A or A/A genotype (26.1% *vs*. 52.0%, *p *= 0.038) and shorter TTP (4.1 months *vs*. 6.2 months, *p *= 0.022). The lower RR with a modified FOLFOX-6 chemotherapy might result from the tendency that the C/C genotype of XPD- Arg156Arg had a higher DNA repair capacity than C/A or A/A genotype [32]. However, it did not affect to prolongation of survival. The other polymorphisms examined failed to show any relation with clinical outcome.

In the presented study, we analyzed germline genotype. Hence the correlation between germline genotype from peripheral blood and somatic genotype from tumor tissues should be considered. Few studies focused on comparing both genotypes. In terms of 28-bp repeat polymorphism, tumor specific loss of heterozygosity at the TS locus has been reported [47]. In contrast, some studies have reported that there is no difference in TS ER polymorphism between genotypes of tumor and normal tissues in gastric [41] and colorectal cancer [40].

Herein, we presume that germline genotypes are nearly identical to somatic genotypes, even though it might not always reflect the tumor genotype. However, germline genotypes determined from peripheral blood mononuclear cells have many strong points. The germline genotypes offer the better clinical accessibility and applicability, compared to tumor tissue DNA or mRNA, which present difficulties in obtaining and handling samples. In our results, germline genotypes, which were obtained by simple blood test, have shown a good association with the clinical outcome and are easily interpreted. In this phase II study, the small sample size (n = 73) might remains a limitation to clarify the role of polymorphism. However, our patient population was considerably homogeneous in terms of ethnicity and chemotherapeutic regimen, and this protocol was prospective.

## Conclusion

We found that modified FOLFOX-6 chemotherapy appears to be effective. A 6-bp deletion homozygote of TS-3'UTR was found to be a predictive marker of the response and was also found to be associated with a prolonged TTP in AGC patients on modified FOLFOX-6 chemotherapy. Moreover, our results suggest that germline genetic polymorphisms of TS and XPD may be useful candidates for a pharmacogenomic prediction of the response to modified FOLFOX-6 chemotherapy in AGC. Alternative treatment other than 5-FU or platinum based therapy should be considered for the patients who have unfavorable genotype. We conclude that testing for the TS-3'UTR 6-bp deletion and XPD polymorphisms might be a candidate pharmacogenomic factors to be explored in the future larger scale study to identify the gastric cancer patients who might benefit from 5-FU based first line chemotherapy.

## Competing interests

The authors declare that they have no competing interests.

## Authors' contributions

BK collected the data, performed the statistical analysis and drafted the manuscript. S–AI designed the concept of this study, performed the statistical analysis with interpretation and approved the final manuscript. S–WH performed the statistical analysis and critically revised the manuscript. HSH carried out the genotyping and management of the samples. D–YO, JHK, S–HL, D–WK, T–YK, DSH and Y–JB performed the chemotherapy for patients and revised the manuscript. All authors read and approved the final manuscript.

## Pre-publication history

The pre-publication history for this paper can be accessed here:


